# Crystal structure and Hirshfeld surface analysis of 2-(1*H*-indol-3-yl)ethanaminium acetate hemihydrate

**DOI:** 10.1107/S2056989019003347

**Published:** 2019-03-15

**Authors:** Balakrishnan Rajeswari, Radhakrishnan Santhi, Palaniyappan Sivajeyanthi, Kasthuri Balasubramani

**Affiliations:** a PG and Research Department of Chemistry, Seethalakshmi Ramaswamy College, Tiruchirappalli-2, Tamil Nadu, India; b Department of Chemistry, Government Arts College (Autonomous), Thanthonimalai, Karur 639 005, Tamil Nadu, India

**Keywords:** crystal structure, 2-(1*H*-indol-3-yl)ethanaminium, acetate, trypamine, hydrogen bonding, Hirshfeld surface analysis

## Abstract

The title mol­ecular salt, C_10_H_13_N_2_^+^·C_2_H_3_O_2_^−^·0.5H_2_O, crystallized with four 2-(1*H*-indol-3-yl)ethanaminium cations (*A*, *B*, *C* and *D*) and four acetate anions in the asymmetric unit, together with two water mol­ecules of crystallization. Each cation is linked to an anion by a C—H⋯π inter­action. The alkyl­aminium side chains have folded conformations, with N—C—C—C torsion angles of −58.5 (3), 59.5 (3), −64.6 (3) and −56.0 (3)° for cations *A*, *B*, *C* and *D*, respectively. In the crystal, the cations and anions are liked by N—H⋯O and C—H⋯O hydrogen bonds, forming chains propagating along the *b*-axis direction. The chains are linked by the water mol­ecules *via* O_water_—H⋯O and N—H⋯O_water_ hydrogen bonds, forming layers lying parallel to the *bc* plane. The overall inter­molecular inter­actions were investigated using Hirshfeld surfaces analysis.

## Abstract 2

The title mol­ecular salt crystallized with four 2-(1*H*-indol-3-yl)ethanaminium cations and four acetate anions in the asymmetric unit, together with two water mol­ecules of crystallization.

## Chemical context   

2-(1*H*-Indol-3-yl)ethanamine (tryptamine) is an alkaloid found in plants and fungi and is a possible inter­mediate in the biosynthetic pathway to the plant hormone indole-3-acetic acid (Takahashi, 1986 ▸). It is also found in trace amounts in the mammalian brain, possibly acting as a neuromodulator or neurotransmitter (Jones, 1982 ▸). As a relatively strong base (p*K*_a_ = 10.2), it readily forms salts with a number of organic acids. There are seven known families of serotonin receptors which are tryptamine derivatives, and all of them are neurotransmitters. Hallucinogens all have a high affinity for certain serotonin receptor subtypes and the relative hallucinogenic potencies of various drugs can be gauged by their affinities for these receptors (Glennon *et al.*, 1984 ▸; Nichols & Sanders-Bush, 2001 ▸; Johnson *et al.*, 1987 ▸; Krebs-Thomson *et al.*, 1998 ▸). The structures of many hallucinogens are similar to serotonin and have a tryptamine core. Indole analogues, especially of tryptamine derivatives, have been found to be polyamine site antagonists at the *N*-methyl­daspartate receptor (Worthen *et al.*, 2001 ▸). Indole and its derivatives are secondary metabolites that are present in most plants (such as unripe bananas, broccoli and cloves), almost all flower oils (jasmine and orange blossoms) and coal tar (Waseem & Mark, 2005 ▸; Lee *et al.*, 2003 ▸). In the pharmaceutical field, it has been discovered that it has anti­microbial and anti-inflammatory properties (Mohammad & Moutaery, 2005 ▸). The title compound, namely 2-(1*H*-indol-3-yl)ethanaminium acetate hemihydrate, was synthesized and its crystal structure and Hirshfeld surface analysis are reported herein.

## Structural commentary   

**Figure 1:**
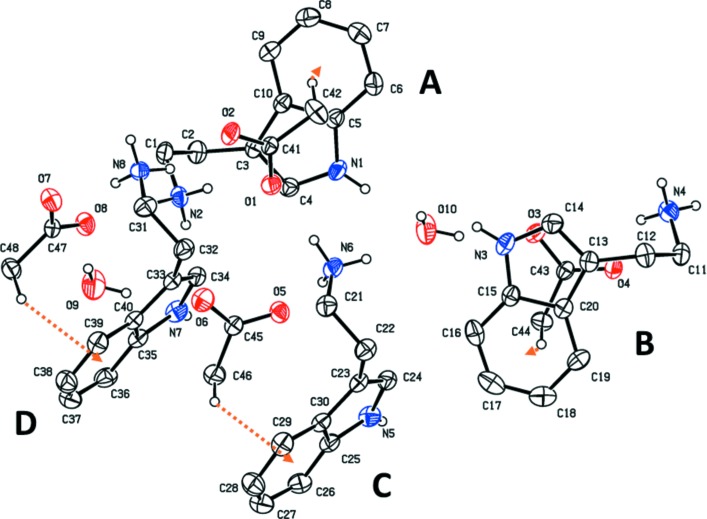
A view of the mol­ecular structure of the title mol­ecular salt, with the atom labelling. Displacement ellipsoids are drawn at the 30% probability level. The C—H⋯π inter­actions linking an anion to a cation are shown as orange arrows (see Table 1 ▸). For clarity, the majority of the C-bound H atoms have been omitted.

The mol­ecular structure of the title salt is shown in Fig. 1 ▸. The asymmetric unit contains four crystallographically independent 2-(1*H*-indol-3-yl)ethanaminium cations, four acetate anions and two water mol­ecules. The cations are protonated at the amine N atoms (N2, N4, N6 and N8) and are each linked to an anion by a C—H⋯π inter­action (Fig. 1 ▸ and Table 1 ▸). The alkyl­aminium side chain in each cation has a folded conformation; the torsion angles are −58.5 (3)° for N2—C1—C2—C3, 59.5 (3)° for N4—C11—C12—C13, −64.6 (3)° for N6—C21—C22—C23 and −56.0 (3)° for N8—C31—C32—C33. These values are similar to those observed in the majority of 2-(1*H*-indol-3-yl)ethanaminium salts (see *Database survey section*, §5). In the structure of tryptamine, determined from powder diffraction data (Nowell *et al.*, 2002 ▸), the corresponding angle is *ca* 60.4°.
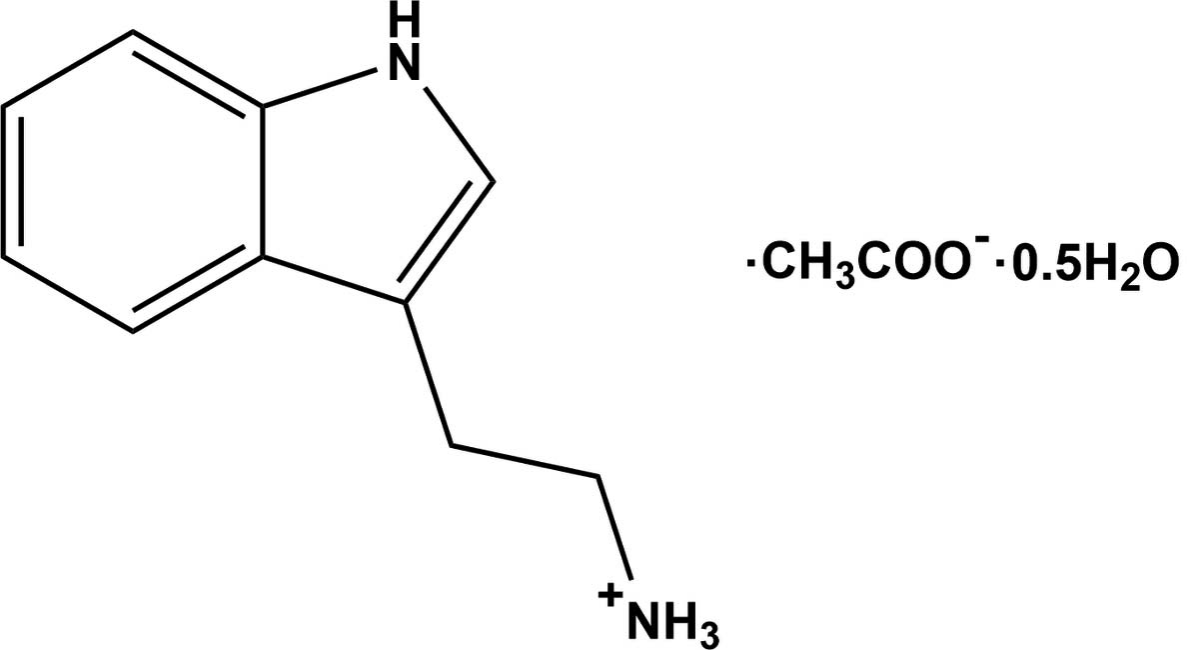


## Supra­molecular features   

**Figure 2:**
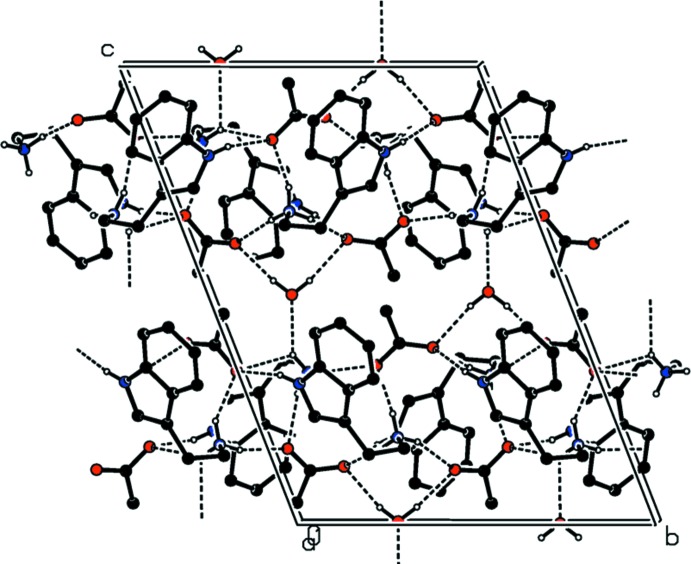
A view along the *a* axis of the crystal packing of the title mol­ecular salt. The N—H⋯O and O—H⋯O hydrogen bonds are shown as dashed lines (see Table 1 ▸). For clarity, the C-bound H atoms have been omitted.

In the crystal, the cations and anions are liked by N—H⋯O and C—H⋯O hydrogen bonds, forming chains propagating along the *b*-axis direction (Fig. 2 ▸ and Table 1 ▸). The chains are linked by the water mol­ecules (O9 and O10) *via* O_water_—H⋯O and N—H⋯O_water_ hydrogen bonds, forming layers lying parallel to the *bc* plane (Fig. 2 ▸ and Table 1 ▸). Within the layers, there are a number of C—H⋯π inter­actions present (Table 1 ▸).

## Hirshfeld surface analysis   

**Figure 3:**
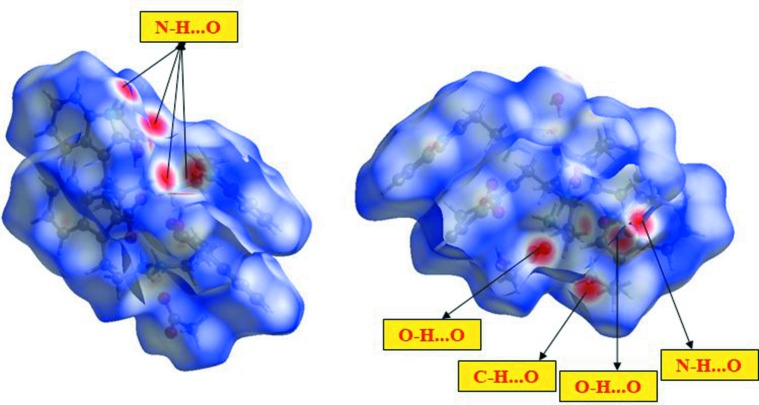
Two views of the overall Hirshfeld surface mapped over *d*_norm_ for the title mol­ecular salt.

**Figure 4:**
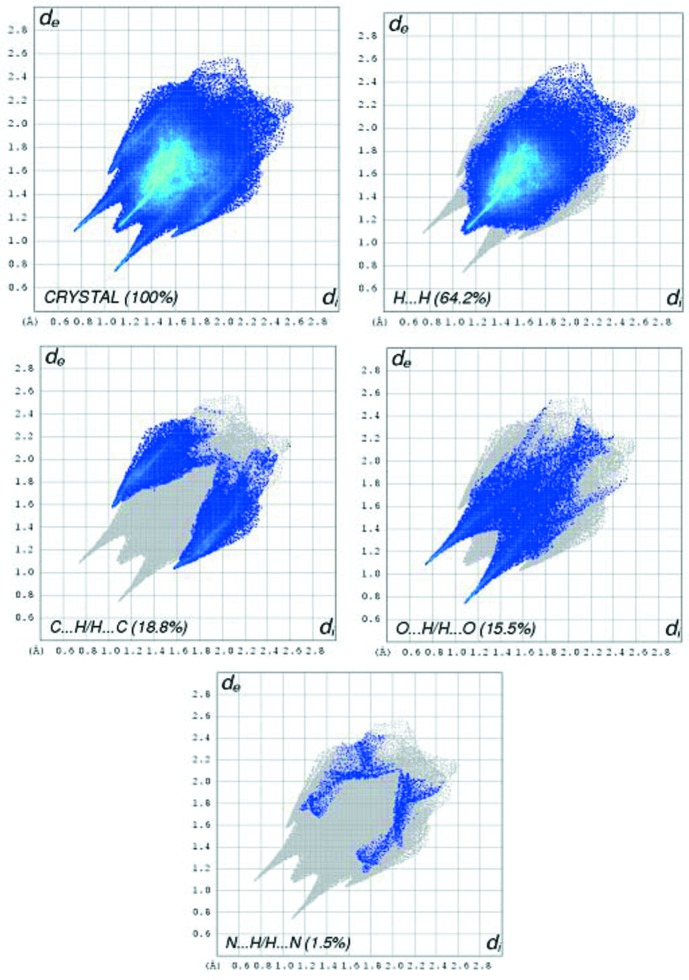
The total two-dimensional fingerprint plot of the crystal and of the relative contributions of the atom pairs to the Hirshfeld surface.

The Hirshfeld surface analysis (Spackman & Jayatilaka, 2009 ▸) and the associated two-dimensional (2D) fingerprint plots (McKinnon *et al.*, 2007 ▸) were performed with *CrystalExplorer17* (Turner *et al.*, 2017 ▸). The Hirshfeld surface of the title mol­ecular salt mapped over *d*_norm_ is given in Fig. 3 ▸. The red points, which represent closer contacts and negative *d*_norm_ values on the surface, correspond to the N—H⋯O, O—H⋯O and C—H⋯O inter­actions. The 2D fingerprint plots are given in Fig. 4 ▸. They reveal that the principal inter­molecular inter­actions are H⋯H (64.2%), C⋯H/H⋯C (18.8%), O⋯H/H⋯O (15.5%) and N⋯H/H⋯N (1.5%), as shown in Fig. 4 ▸.

## Database survey   

A search of the Cambridge Structural Database (CSD, Version 5.40, update November 2018; Groom *et al.*, 2016 ▸) for 2-(1*H*-indol-3-yl)ethanamines yielded 42 hits for structures that include atomic coordinates. In 14 hits, the alkyl­aminium side chain has an extended conformation, with the absolute value of the N—C—C—C torsion angle varying from *ca* 169.69° in the thio­phene-2-carboxyl­ate salt (CSD refcode LACPUA; Koshima & Honke, 1999 ▸) to *ca* 179.44° in the (2*S*,3*S*)-hydrogen tartrate monohydrate salt (SOCMED; Koleva *et al.*, 2009 ▸). In 28 hits, the alkyl­aminium side chain has a folded conformation as in the title cations. For example, in the di­phenyl­acetate salt (WODVUG; Koshima *et al.*, 1999 ▸), the torsion angle is *ca* 64.38°, or for the chloride salt (TRYPTA11; Parsons *et al.*, 2015 ▸), the torsion angle is *ca* −59.43°. An analysis showed that only three compounds crystallize with *Z*′ > 1. They are tris­(tryptaminium) tris­(3,5-di­nitro­benzoate) bis­(quinoline) dihydrate (AWIDAN; Lynch *et al.*, 2016 ▸), with *Z*′ = 3, the benzoate salt (DAMNAH; Terakita *et al.*, 2004 ▸), with *Z*′ = 2, and (cucurbit[6]uril) bis­(tryptamine) dichloride penta­deca­hydrate (DASSOH; Danylyuk & Fedin, 2012 ▸), also with *Z*′ = 2. In DAMNAH, the alkyl­aminium side chain has a folded conformation, while in the other two compounds the side chain is extended.

## Synthesis and crystallization   

The title compound was synthesized by the reaction of a 1:1 stoichiometric mixture of tryptamine (0.160 mg, Aldrich) and acetic acid (0.060 mg, Merck) in a hot methano­lic solution (20 ml) with 10 ml of water. After warming for a few minutes over a water bath, the solution was cooled and kept at room temperature. Within a few days, colourless needle-like crystals, suitable for the X-ray analysis, were obtained (yield 65%).

## Refinement   

Crystal data, data collection and structure refinement details are summarized in Table 2 ▸. The water O-bound H atoms were located in a difference Fourier map and freely refined. The NH and NH_3_ hydrogens were originally located in a difference Fourier map but for refinement, together with the C-bound H atoms, they were positioned geometrically and refined using a riding model, with N—H = 0.86–0.89 Å and C—H = 0.93–0.97 Å, and with *U*_iso_(H) = 1.5*U*_eq_(C,N) for methyl and aminium H atoms, and 1.2*U*_eq_(C,N) otherwise. The structure was refined as a two-component twin with twin law (02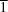); BASF = 0.074 (1).

## Supplementary Material

Crystal structure: contains datablock(s) global, I. DOI: 10.1107/S2056989019003347/su5480sup1.cif

Structure factors: contains datablock(s) I. DOI: 10.1107/S2056989019003347/su5480Isup2.hkl

CCDC reference: 1579740

Additional supporting information:  crystallographic information; 3D view; checkCIF report

## Figures and Tables

**Table 1:** Hydrogen-bond geometry (Å, °) *Cg*2, *Cg*5, *Cg*8 and *Cg*11 are the centroids of the benzene rings C5–C10, C15–C20, C25–C30 and C35–C40, respectively. *Cg*3, *Cg*6, *Cg*9 and *Cg*12 are the centroids of the indole ring systems N1/C3–C10, N3/C13–C20, N5/C23–C30 and N7/C33–C40, respectively.

*D*—H⋯*A*	*D*—H	H⋯*A*	*D*⋯*A*	*D*—H⋯*A*
C42—H42*B*⋯*Cg*2	0.96	2.87	3.621 (3)	135
C44—H44*B*⋯*Cg*5	0.96	2.74	3.550 (3)	143
C46—H46*B*⋯*Cg*8	0.96	2.80	3.533 (3)	134
C48—H48*B*⋯*Cg*11	0.96	2.78	3.629 (3)	147
N1—H1*N*⋯O4^i^	0.86	2.08	2.898 (2)	159
N2—H2*AN*⋯O6	0.89	2.02	2.861 (3)	156
N2—H2*BN*⋯O8	0.89	1.93	2.778 (2)	158
N2—H2*CN*⋯O1	0.89	1.92	2.803 (2)	169
N3—H3*N*⋯O2^ii^	0.86	2.04	2.864 (3)	161
N4—H4*AN*⋯O4	0.89	2.03	2.805 (3)	145
N4—H4*BN*⋯O5^i^	0.89	1.91	2.777 (2)	163
N4—H4*CN*⋯O7^ii^	0.89	2.45	3.186 (3)	140
N5—H5*N*⋯O7^iii^	0.86	2.01	2.839 (2)	162
N6—H6*AN*⋯O4^i^	0.89	2.57	3.122 (3)	121
N6—H6*BN*⋯O1	0.89	1.95	2.828 (2)	169
N6—H6*CN*⋯O5	0.89	2.07	2.936 (3)	165
N7—H7*N*⋯O6	0.86	2.04	2.867 (3)	161
N8—H8*AN*⋯O2	0.89	2.09	2.936 (3)	157
N8—H8*BN*⋯O3^ii^	0.89	1.85	2.734 (2)	172
N8—H8*CN*⋯O7	0.89	1.87	2.726 (2)	162
C4—H4⋯O5	0.93	2.40	3.248 (3)	151
C34—H34⋯O1	0.93	2.46	3.347 (3)	159
N4—H4*CN*⋯O9^iv^	0.89	2.46	3.003 (3)	120
O9—H9*A*⋯O8	0.88 (5)	1.97 (5)	2.840 (3)	169 (4)
O9—H9*B*⋯O6	0.86 (4)	2.02 (4)	2.872 (3)	168 (4)
N6—H6*AN*⋯O10	0.89	2.22	2.927 (3)	136
O10—H10*A*⋯O3	0.88 (4)	1.96 (4)	2.822 (3)	166 (3)
O10—H10*B*⋯O2^ii^	0.85 (4)	2.07 (4)	2.903 (3)	169 (3)
C9—H9⋯*Cg*12^v^	0.93	2.93	3.782 (3)	153
C19—H19⋯*Cg*9^vi^	0.93	2.81	3.641 (3)	149
C29—H29⋯*Cg*3^vii^	0.93	2.92	3.736 (3)	147
C39—H39⋯*Cg*6^viii^	0.96	2.95	3.643 (3)	132

Symmetry codes: (i) 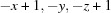; (ii) 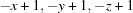; (iii) 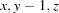; (iv) 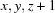; (v) 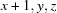; (vi) 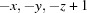; (vii) 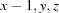; (viii) 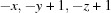.

**Table 2:** Experimental details

Crystal data	
Chemical formula	C_10_H_13_N_2_^+^·C_2_H_3_O_2_^−^·0.5H_2_O
*M*_r_	229.27
Crystal system, space group	Triclinic, *P* 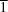
Temperature (K)	296
*a*, *b*, *c* (Å)	10.8328 (2), 13.2452 (2), 18.1426 (3)
α, β, γ (°)	111.276 (1), 90.182 (1), 90.125 (1)
*V* (Å^3^)	2425.70 (7)
*Z*	8
Radiation type	Cu *K*α
μ (mm^−1^)	0.72
Crystal size (mm)	0.10 × 0.10 × 0.05

Data collection	
Diffractometer	Bruker Kappa APEXIII CMOS
Absorption correction	Multi-scan (*SADABS*; Bruker, 2016 ▸)
*T*_min_, *T*_max_	0.705, 0.754
No. of measured, independent and observed [*I* > 2σ(*I*)] reflections	49248, 9420, 6239
*R*_int_	0.069
(sin θ/λ)_max_ (Å^−1^)	0.619

Refinement	
*R*[*F* ^2^ > 2σ(*F* ^2^)], *wR*(*F* ^2^), *S*	0.054, 0.142, 1.06
No. of reflections	9420
No. of parameters	620
H-atom treatment	H atoms treated by a mixture of independent and constrained refinement
Δρ_max_, Δρ_min_ (e Å^−3^)	0.22, −0.23

Computer programs: *APEX3* (Bruker, 2016 ▸), *SAINT* (Bruker, 2016 ▸), *SAINT*/*XPREP* (Bruker, 2016 ▸), *SHELXL2018* (Sheldrick, 2015*a* ▸), *SHELXL2018* (Sheldrick, 2015*b* ▸) and *PLATON* (Spek, 2009 ▸).

## supplementary crystallographic information

### Crystal data

**Table:**

C_10_H_13_N_2_^+^·C_2_H_3_O_2_^−^·0.5H_2_O	*Z* = 8
*M**_r_* = 229.27	*F*(000) = 984
Triclinic, *P*1	*D*_x_ = 1.256 Mg m^−^^3^
*a* = 10.8328 (2) Å	Cu *K*α radiation, λ = 1.54178 Å
*b* = 13.2452 (2) Å	Cell parameters from 9851 reflections
*c* = 18.1426 (3) Å	θ = 3.6–72.4°
α = 111.276 (1)°	µ = 0.72 mm^−^^1^
β = 90.182 (1)°	*T* = 296 K
γ = 90.125 (1)°	Needle, yellow
*V* = 2425.70 (7) Å^3^	0.10 × 0.10 × 0.05 mm

### Data collection

**Table:**

Bruker Kappa APEXIII CMOS diffractometer	6239 reflections with *I* > 2σ(*I*)
Radiation source: micro-focus sealed tube	*R*_int_ = 0.069
ω and φ scan	θ_max_ = 72.6°, θ_min_ = 2.6°
Absorption correction: multi-scan (SADABS; Bruker, 2016)	*h* = −13→13
*T*_min_ = 0.705, *T*_max_ = 0.754	*k* = −16→15
49248 measured reflections	*l* = −21→22
9420 independent reflections	

### Refinement

**Table:**

Refinement on *F*^2^	Primary atom site location: structure-invariant direct methods
Least-squares matrix: full	Secondary atom site location: difference Fourier map
*R*[*F*^2^ > 2σ(*F*^2^)] = 0.054	Hydrogen site location: mixed
*wR*(*F*^2^) = 0.142	H atoms treated by a mixture of independent and constrained refinement
*S* = 1.06	*w* = 1/[σ^2^(*F*_o_^2^) + (0.0577*P*)^2^ + 0.6703*P*] where *P* = (*F*_o_^2^ + 2*F*_c_^2^)/3
9420 reflections	(Δ/σ)_max_ = 0.001
620 parameters	Δρ_max_ = 0.22 e Å^−^^3^
0 restraints	Δρ_min_ = −0.23 e Å^−^^3^

### Special details

**Table:**

Geometry. All esds (except the esd in the dihedral angle between two l.s. planes) are estimated using the full covariance matrix. The cell esds are taken into account individually in the estimation of esds in distances, angles and torsion angles; correlations between esds in cell parameters are only used when they are defined by crystal symmetry. An approximate (isotropic) treatment of cell esds is used for estimating esds involving l.s. planes.
Refinement. Refined as a 2-component twin. BASF = 0.074 (1)

### Fractional atomic coordinates and isotropic or equivalent isotropic displacement parameters (Å^2^)

**Table:**

	*x*	*y*	*z*	*U*_iso_*/*U*_eq_	
N1	0.7460 (2)	0.15380 (15)	0.30549 (12)	0.0469 (5)	
H1N	0.712448	0.108918	0.323851	0.056*	
N2	0.58562 (19)	0.36678 (15)	0.18666 (11)	0.0417 (5)	
H2AN	0.556481	0.300879	0.159185	0.063*	
H2BN	0.540279	0.416060	0.176314	0.063*	
H2CN	0.582104	0.378500	0.238110	0.063*	
C1	0.7162 (2)	0.3750 (2)	0.16382 (15)	0.0485 (6)	
H1A	0.756250	0.434967	0.204862	0.058*	
H1B	0.717643	0.389992	0.115316	0.058*	
C2	0.7870 (3)	0.2718 (2)	0.15173 (14)	0.0478 (6)	
H2A	0.749936	0.213332	0.108028	0.057*	
H2B	0.871094	0.281055	0.137000	0.057*	
C3	0.7902 (2)	0.23981 (17)	0.22285 (13)	0.0370 (5)	
C4	0.7217 (2)	0.16035 (18)	0.23366 (14)	0.0427 (6)	
H4	0.665741	0.116330	0.196956	0.051*	
C5	0.8327 (2)	0.23072 (18)	0.34366 (14)	0.0393 (5)	
C6	0.8882 (3)	0.2558 (2)	0.41718 (15)	0.0511 (7)	
H6	0.867764	0.218304	0.449981	0.061*	
C7	0.9740 (3)	0.3378 (2)	0.44000 (17)	0.0581 (7)	
H7	1.011505	0.356765	0.489394	0.070*	
C8	1.0061 (3)	0.3931 (2)	0.39045 (17)	0.0594 (7)	
H8	1.065694	0.447436	0.407008	0.071*	
C9	0.9515 (2)	0.3691 (2)	0.31801 (16)	0.0500 (6)	
H9	0.973393	0.406875	0.285679	0.060*	
C10	0.8626 (2)	0.28713 (17)	0.29309 (14)	0.0378 (5)	
N3	0.2703 (2)	0.33098 (16)	0.69103 (13)	0.0488 (5)	
H3N	0.303553	0.371522	0.668745	0.059*	
N4	0.4274 (2)	0.13833 (15)	0.82987 (12)	0.0486 (5)	
H4AN	0.432242	0.124335	0.778135	0.073*	
H4BN	0.469902	0.088765	0.841737	0.073*	
H4CN	0.458857	0.203729	0.856258	0.073*	
C11	0.2965 (3)	0.13525 (19)	0.85230 (15)	0.0477 (6)	
H11A	0.292666	0.126283	0.902982	0.057*	
H11B	0.256054	0.073273	0.813351	0.057*	
C12	0.2288 (3)	0.2371 (2)	0.85778 (14)	0.0498 (7)	
H12A	0.144212	0.231717	0.873460	0.060*	
H12B	0.266920	0.298267	0.898861	0.060*	
C13	0.2276 (2)	0.25876 (17)	0.78232 (14)	0.0398 (6)	
C14	0.2985 (2)	0.33206 (18)	0.76495 (15)	0.0461 (6)	
H14	0.357747	0.376541	0.798494	0.055*	
C15	0.1806 (2)	0.25507 (18)	0.65849 (14)	0.0411 (6)	
C16	0.1220 (3)	0.2242 (2)	0.58509 (16)	0.0568 (7)	
H16	0.143241	0.255170	0.548317	0.068*	
C17	0.0315 (3)	0.1463 (3)	0.56926 (18)	0.0677 (9)	
H17	−0.009486	0.124404	0.520839	0.081*	
C18	−0.0002 (3)	0.0995 (2)	0.62359 (18)	0.0654 (8)	
H18	−0.062175	0.047218	0.611065	0.078*	
C19	0.0581 (3)	0.1290 (2)	0.69548 (16)	0.0522 (7)	
H19	0.036057	0.096834	0.731362	0.063*	
C20	0.1507 (2)	0.20764 (18)	0.71446 (13)	0.0383 (5)	
N5	0.2644 (2)	−0.14717 (15)	0.19676 (12)	0.0475 (5)	
H5N	0.296545	−0.210710	0.177804	0.057*	
N6	0.4361 (2)	0.18460 (15)	0.32742 (12)	0.0474 (5)	
H6AN	0.461588	0.161044	0.365009	0.071*	
H6BN	0.480121	0.242580	0.329878	0.071*	
H6CN	0.446204	0.132574	0.280243	0.071*	
C21	0.3040 (2)	0.21386 (18)	0.33925 (15)	0.0467 (6)	
H21A	0.272952	0.227723	0.293620	0.056*	
H21B	0.295285	0.279873	0.385235	0.056*	
C22	0.2279 (3)	0.12453 (19)	0.35085 (14)	0.0493 (6)	
H22A	0.261412	0.109324	0.395359	0.059*	
H22B	0.144073	0.150151	0.364031	0.059*	
C23	0.2246 (2)	0.02147 (17)	0.28001 (14)	0.0396 (5)	
C24	0.2920 (2)	−0.06968 (19)	0.26856 (15)	0.0444 (6)	
H24	0.348676	−0.077948	0.304533	0.053*	
C25	0.1777 (2)	−0.10725 (18)	0.16006 (14)	0.0410 (6)	
C26	0.1195 (3)	−0.1548 (2)	0.08716 (16)	0.0547 (7)	
H26	0.138459	−0.225136	0.054130	0.066*	
C27	0.0334 (3)	−0.0951 (2)	0.06532 (18)	0.0649 (8)	
H27	−0.006303	−0.125367	0.016656	0.078*	
C28	0.0043 (3)	0.0098 (3)	0.11456 (19)	0.0646 (8)	
H28	−0.055184	0.048063	0.098487	0.077*	
C29	0.0618 (3)	0.0581 (2)	0.18678 (17)	0.0531 (7)	
H29	0.041862	0.128457	0.219234	0.064*	
C30	0.1508 (2)	−0.00045 (17)	0.21039 (14)	0.0392 (5)	
N7	0.2593 (2)	0.35813 (15)	0.18889 (13)	0.0482 (5)	
H7N	0.294125	0.296121	0.167137	0.058*	
N8	0.40770 (19)	0.69036 (15)	0.32048 (11)	0.0430 (5)	
H8AN	0.438094	0.640145	0.337327	0.065*	
H8BN	0.453345	0.750285	0.339608	0.065*	
H8CN	0.409079	0.665847	0.267862	0.065*	
C31	0.2781 (3)	0.71517 (19)	0.34835 (15)	0.0483 (6)	
H31A	0.233100	0.740203	0.311949	0.058*	
H31B	0.278233	0.773101	0.399827	0.058*	
C32	0.2130 (3)	0.6172 (2)	0.35426 (14)	0.0498 (6)	
H32A	0.252725	0.597867	0.395266	0.060*	
H32B	0.128300	0.636587	0.370354	0.060*	
C33	0.2123 (2)	0.51998 (17)	0.27897 (13)	0.0375 (5)	
C34	0.2853 (2)	0.43123 (19)	0.26256 (15)	0.0448 (6)	
H34	0.344421	0.421696	0.296698	0.054*	
C35	0.1695 (2)	0.39916 (18)	0.15541 (14)	0.0398 (5)	
C36	0.1129 (3)	0.3559 (2)	0.08117 (15)	0.0531 (7)	
H36	0.136157	0.288782	0.044803	0.064*	
C37	0.0219 (3)	0.4157 (3)	0.06369 (17)	0.0623 (8)	
H37	−0.017469	0.388519	0.014738	0.075*	
C38	−0.0125 (3)	0.5161 (2)	0.11781 (17)	0.0601 (7)	
H38	−0.074693	0.554782	0.104500	0.072*	
C39	0.0436 (2)	0.5595 (2)	0.19055 (16)	0.0495 (6)	
H39	0.019483	0.626784	0.226234	0.059*	
C40	0.1370 (2)	0.50153 (17)	0.21034 (13)	0.0357 (5)	
O1	0.54686 (17)	0.38518 (13)	0.34369 (10)	0.0480 (4)	
O2	0.56930 (18)	0.56213 (13)	0.38279 (11)	0.0557 (5)	
C41	0.5852 (2)	0.47330 (18)	0.39205 (13)	0.0359 (5)	
C42	0.6554 (3)	0.4733 (2)	0.46310 (15)	0.0605 (8)	
H42A	0.635115	0.537044	0.507692	0.091*	
H42B	0.742370	0.472921	0.452932	0.091*	
H42C	0.633916	0.410031	0.474307	0.091*	
O3	0.46376 (17)	0.12110 (13)	0.60828 (10)	0.0495 (4)	
O4	0.40630 (17)	0.01048 (13)	0.66873 (9)	0.0482 (4)	
C43	0.4023 (2)	0.04290 (16)	0.61187 (13)	0.0350 (5)	
C44	0.3194 (3)	−0.0161 (2)	0.54327 (14)	0.0501 (6)	
H44A	0.341903	0.002369	0.498563	0.075*	
H44B	0.235268	0.004489	0.557315	0.075*	
H44C	0.327943	−0.092742	0.530034	0.075*	
O5	0.46117 (17)	0.04508 (13)	0.16035 (10)	0.0478 (4)	
O6	0.42758 (18)	0.18212 (13)	0.12116 (11)	0.0572 (5)	
C45	0.4181 (2)	0.08336 (17)	0.11236 (13)	0.0368 (5)	
C46	0.3498 (3)	0.0099 (2)	0.04132 (15)	0.0581 (7)	
H46A	0.371962	0.027056	−0.004017	0.087*	
H46B	0.262618	0.019558	0.050183	0.087*	
H46C	0.371140	−0.063933	0.032166	0.087*	
O7	0.40477 (17)	0.66077 (12)	0.16375 (9)	0.0493 (5)	
O8	0.46056 (17)	0.48909 (12)	0.11445 (10)	0.0497 (5)	
C47	0.4017 (2)	0.56810 (17)	0.11097 (13)	0.0341 (5)	
C48	0.3219 (3)	0.5520 (2)	0.03979 (14)	0.0508 (7)	
H48A	0.326314	0.615251	0.025871	0.076*	
H48B	0.238031	0.540220	0.051669	0.076*	
H48C	0.350369	0.490185	−0.003708	0.076*	
O9	0.4626 (3)	0.2741 (2)	0.00169 (13)	0.0817 (8)	
H9A	0.455 (4)	0.342 (4)	0.033 (3)	0.136 (18)*	
H9B	0.449 (4)	0.238 (3)	0.032 (2)	0.107 (15)*	
O10	0.4565 (3)	0.2236 (2)	0.49644 (13)	0.0767 (7)	
H10A	0.447 (3)	0.187 (3)	0.527 (2)	0.094 (12)*	
H10B	0.442 (3)	0.288 (3)	0.527 (2)	0.091 (13)*	

### Atomic displacement parameters (Å^2^)

**Table:**

	*U*^11^	*U*^22^	*U*^33^	*U*^12^	*U*^13^	*U*^23^
N1	0.0499 (14)	0.0404 (11)	0.0599 (14)	−0.0059 (9)	−0.0003 (10)	0.0296 (10)
N2	0.0536 (14)	0.0351 (10)	0.0381 (11)	0.0032 (9)	−0.0046 (9)	0.0154 (9)
C1	0.0576 (18)	0.0490 (14)	0.0486 (15)	−0.0009 (12)	0.0012 (12)	0.0294 (12)
C2	0.0546 (18)	0.0465 (14)	0.0435 (14)	0.0068 (12)	0.0079 (12)	0.0175 (12)
C3	0.0398 (15)	0.0313 (11)	0.0418 (13)	0.0062 (9)	0.0053 (10)	0.0152 (10)
C4	0.0428 (16)	0.0356 (12)	0.0492 (15)	−0.0014 (10)	−0.0028 (11)	0.0148 (11)
C5	0.0368 (15)	0.0351 (12)	0.0501 (14)	0.0036 (10)	0.0040 (11)	0.0204 (11)
C6	0.0561 (19)	0.0534 (16)	0.0516 (16)	0.0062 (13)	0.0005 (13)	0.0280 (13)
C7	0.052 (2)	0.0632 (18)	0.0568 (17)	0.0035 (14)	−0.0099 (13)	0.0194 (14)
C8	0.054 (2)	0.0535 (16)	0.0666 (19)	−0.0137 (13)	−0.0072 (14)	0.0170 (14)
C9	0.0482 (18)	0.0444 (14)	0.0597 (17)	−0.0087 (11)	0.0045 (13)	0.0218 (13)
C10	0.0367 (14)	0.0324 (12)	0.0463 (13)	0.0047 (9)	0.0071 (10)	0.0169 (10)
N3	0.0525 (15)	0.0417 (12)	0.0615 (14)	−0.0002 (10)	0.0090 (11)	0.0296 (10)
N4	0.0584 (15)	0.0350 (11)	0.0520 (13)	0.0050 (9)	−0.0072 (10)	0.0154 (9)
C11	0.0624 (19)	0.0430 (14)	0.0420 (14)	0.0011 (12)	0.0011 (12)	0.0207 (11)
C12	0.0600 (19)	0.0484 (15)	0.0400 (14)	0.0129 (12)	0.0065 (12)	0.0148 (12)
C13	0.0430 (15)	0.0322 (12)	0.0437 (13)	0.0099 (10)	0.0051 (11)	0.0131 (10)
C14	0.0462 (17)	0.0324 (12)	0.0577 (16)	0.0029 (10)	−0.0016 (12)	0.0138 (11)
C15	0.0401 (15)	0.0390 (13)	0.0482 (14)	0.0084 (10)	0.0069 (11)	0.0208 (11)
C16	0.059 (2)	0.0687 (19)	0.0502 (16)	0.0180 (15)	0.0014 (13)	0.0307 (14)
C17	0.054 (2)	0.078 (2)	0.0608 (19)	0.0103 (16)	−0.0148 (15)	0.0130 (16)
C18	0.050 (2)	0.0666 (19)	0.071 (2)	−0.0087 (14)	−0.0037 (15)	0.0148 (16)
C19	0.0457 (18)	0.0509 (15)	0.0606 (17)	−0.0052 (12)	0.0125 (13)	0.0210 (13)
C20	0.0366 (15)	0.0358 (12)	0.0430 (13)	0.0066 (10)	0.0087 (10)	0.0149 (10)
N5	0.0509 (14)	0.0278 (10)	0.0604 (14)	0.0041 (9)	−0.0006 (10)	0.0121 (10)
N6	0.0567 (15)	0.0351 (11)	0.0529 (13)	−0.0059 (9)	−0.0018 (10)	0.0192 (10)
C21	0.0594 (19)	0.0310 (12)	0.0456 (14)	0.0023 (11)	0.0013 (12)	0.0089 (11)
C22	0.0552 (18)	0.0466 (14)	0.0426 (14)	−0.0015 (12)	0.0104 (12)	0.0119 (12)
C23	0.0427 (15)	0.0335 (12)	0.0443 (14)	−0.0038 (10)	0.0058 (11)	0.0160 (10)
C24	0.0448 (16)	0.0405 (13)	0.0521 (15)	−0.0018 (11)	−0.0022 (11)	0.0215 (12)
C25	0.0379 (15)	0.0326 (12)	0.0530 (15)	−0.0015 (10)	0.0035 (11)	0.0162 (11)
C26	0.056 (2)	0.0452 (15)	0.0574 (17)	−0.0081 (12)	−0.0026 (13)	0.0114 (13)
C27	0.059 (2)	0.073 (2)	0.0607 (18)	−0.0162 (15)	−0.0139 (14)	0.0228 (16)
C28	0.050 (2)	0.075 (2)	0.076 (2)	0.0086 (15)	−0.0085 (15)	0.0365 (17)
C29	0.0482 (18)	0.0483 (15)	0.0655 (18)	0.0134 (12)	0.0110 (14)	0.0237 (13)
C30	0.0358 (15)	0.0347 (12)	0.0486 (14)	−0.0001 (9)	0.0074 (11)	0.0168 (10)
N7	0.0509 (14)	0.0296 (10)	0.0623 (14)	0.0067 (9)	0.0055 (10)	0.0146 (10)
N8	0.0534 (14)	0.0361 (10)	0.0387 (11)	−0.0081 (9)	−0.0069 (9)	0.0126 (9)
C31	0.0550 (18)	0.0375 (13)	0.0440 (14)	−0.0028 (11)	0.0053 (12)	0.0046 (11)
C32	0.0594 (19)	0.0506 (15)	0.0376 (14)	−0.0083 (12)	0.0059 (12)	0.0140 (12)
C33	0.0386 (15)	0.0343 (12)	0.0419 (13)	−0.0050 (9)	0.0040 (10)	0.0167 (10)
C34	0.0437 (16)	0.0445 (14)	0.0538 (15)	−0.0034 (11)	−0.0047 (11)	0.0270 (12)
C35	0.0386 (15)	0.0332 (12)	0.0474 (14)	−0.0019 (10)	0.0056 (11)	0.0146 (11)
C36	0.058 (2)	0.0460 (15)	0.0479 (15)	−0.0137 (12)	0.0020 (13)	0.0085 (12)
C37	0.057 (2)	0.078 (2)	0.0538 (17)	−0.0192 (15)	−0.0137 (14)	0.0265 (16)
C38	0.0467 (19)	0.075 (2)	0.0681 (19)	0.0042 (14)	−0.0044 (14)	0.0377 (16)
C39	0.0463 (17)	0.0464 (14)	0.0587 (17)	0.0109 (12)	0.0085 (13)	0.0223 (13)
C40	0.0361 (14)	0.0315 (11)	0.0409 (13)	−0.0005 (9)	0.0070 (10)	0.0146 (10)
O1	0.0616 (13)	0.0366 (9)	0.0447 (10)	−0.0074 (8)	−0.0071 (8)	0.0136 (8)
O2	0.0727 (14)	0.0408 (10)	0.0631 (12)	−0.0089 (9)	−0.0059 (9)	0.0303 (9)
C41	0.0383 (15)	0.0369 (13)	0.0344 (12)	−0.0009 (9)	0.0039 (10)	0.0151 (10)
C42	0.066 (2)	0.0651 (18)	0.0444 (15)	0.0148 (14)	−0.0101 (13)	0.0130 (13)
O3	0.0599 (13)	0.0397 (9)	0.0521 (10)	−0.0162 (8)	−0.0051 (8)	0.0206 (8)
O4	0.0597 (13)	0.0491 (10)	0.0419 (10)	−0.0083 (8)	−0.0024 (8)	0.0238 (8)
C43	0.0412 (15)	0.0262 (11)	0.0368 (12)	0.0004 (9)	0.0044 (10)	0.0104 (9)
C44	0.0546 (18)	0.0466 (14)	0.0445 (14)	−0.0088 (12)	−0.0093 (12)	0.0111 (12)
O5	0.0568 (12)	0.0386 (9)	0.0497 (10)	0.0028 (8)	−0.0063 (8)	0.0178 (8)
O6	0.0701 (14)	0.0303 (9)	0.0703 (13)	0.0047 (8)	−0.0038 (10)	0.0171 (8)
C45	0.0389 (15)	0.0298 (12)	0.0404 (13)	0.0051 (9)	0.0049 (10)	0.0113 (10)
C46	0.064 (2)	0.0612 (17)	0.0484 (16)	−0.0147 (14)	−0.0071 (13)	0.0192 (13)
O7	0.0664 (13)	0.0353 (9)	0.0394 (9)	0.0087 (8)	−0.0063 (8)	0.0054 (7)
O8	0.0616 (13)	0.0377 (9)	0.0526 (10)	0.0088 (8)	−0.0049 (8)	0.0198 (8)
C47	0.0395 (14)	0.0321 (12)	0.0330 (12)	0.0037 (9)	0.0034 (9)	0.0145 (10)
C48	0.0576 (19)	0.0534 (16)	0.0427 (14)	−0.0003 (12)	−0.0077 (12)	0.0192 (12)
O9	0.138 (2)	0.0510 (14)	0.0508 (13)	−0.0057 (13)	−0.0047 (13)	0.0119 (11)
O10	0.129 (2)	0.0563 (14)	0.0483 (13)	0.0027 (13)	−0.0048 (12)	0.0236 (12)

### Geometric parameters (Å, º)

**Table:**

N1—C4	1.362 (3)	C23—C30	1.430 (3)
N1—C5	1.372 (3)	C24—H24	0.9300
N1—H1N	0.8600	C25—C26	1.390 (4)
N2—C1	1.490 (3)	C25—C30	1.409 (3)
N2—H2AN	0.8900	C26—C27	1.372 (4)
N2—H2BN	0.8900	C26—H26	0.9300
N2—H2CN	0.8900	C27—C28	1.389 (4)
C1—C2	1.514 (3)	C27—H27	0.9300
C1—H1A	0.9700	C28—C29	1.378 (4)
C1—H1B	0.9700	C28—H28	0.9300
C2—C3	1.498 (3)	C29—C30	1.398 (3)
C2—H2A	0.9700	C29—H29	0.9300
C2—H2B	0.9700	N7—C35	1.360 (3)
C3—C4	1.358 (3)	N7—C34	1.364 (3)
C3—C10	1.429 (3)	N7—H7N	0.8600
C4—H4	0.9300	N8—C31	1.490 (3)
C5—C6	1.387 (3)	N8—H8AN	0.8900
C5—C10	1.415 (3)	N8—H8BN	0.8900
C6—C7	1.372 (4)	N8—H8CN	0.8900
C6—H6	0.9300	C31—C32	1.514 (3)
C7—C8	1.395 (4)	C31—H31A	0.9700
C7—H7	0.9300	C31—H31B	0.9700
C8—C9	1.367 (4)	C32—C33	1.499 (3)
C8—H8	0.9300	C32—H32A	0.9700
C9—C10	1.395 (3)	C32—H32B	0.9700
C9—H9	0.9300	C33—C34	1.359 (3)
N3—C15	1.365 (3)	C33—C40	1.431 (3)
N3—C14	1.370 (3)	C34—H34	0.9300
N3—H3N	0.8600	C35—C36	1.397 (3)
N4—C11	1.481 (3)	C35—C40	1.407 (3)
N4—H4AN	0.8900	C36—C37	1.373 (4)
N4—H4BN	0.8900	C36—H36	0.9300
N4—H4CN	0.8900	C37—C38	1.389 (4)
C11—C12	1.509 (3)	C37—H37	0.9300
C11—H11A	0.9700	C38—C39	1.372 (4)
C11—H11B	0.9700	C38—H38	0.9300
C12—C13	1.497 (3)	C39—C40	1.394 (3)
C12—H12A	0.9700	C39—H39	0.9300
C12—H12B	0.9700	O1—C41	1.248 (3)
C13—C14	1.361 (3)	O2—C41	1.259 (3)
C13—C20	1.433 (3)	C41—C42	1.495 (3)
C14—H14	0.9300	C42—H42A	0.9600
C15—C16	1.394 (4)	C42—H42B	0.9600
C15—C20	1.412 (3)	C42—H42C	0.9600
C16—C17	1.374 (4)	O3—C43	1.252 (3)
C16—H16	0.9300	O4—C43	1.254 (3)
C17—C18	1.385 (4)	C43—C44	1.500 (3)
C17—H17	0.9300	C44—H44A	0.9600
C18—C19	1.370 (4)	C44—H44B	0.9600
C18—H18	0.9300	C44—H44C	0.9600
C19—C20	1.394 (3)	O5—C45	1.246 (3)
C19—H19	0.9300	O6—C45	1.263 (3)
N5—C25	1.364 (3)	C45—C46	1.495 (3)
N5—C24	1.366 (3)	C46—H46A	0.9600
N5—H5N	0.8600	C46—H46B	0.9600
N6—C21	1.480 (3)	C46—H46C	0.9600
N6—H6AN	0.8900	O7—C47	1.253 (3)
N6—H6BN	0.8900	O8—C47	1.247 (3)
N6—H6CN	0.8900	C47—C48	1.501 (3)
C21—C22	1.517 (3)	C48—H48A	0.9600
C21—H21A	0.9700	C48—H48B	0.9600
C21—H21B	0.9700	C48—H48C	0.9600
C22—C23	1.498 (3)	O9—H9A	0.88 (5)
C22—H22A	0.9700	O9—H9B	0.86 (4)
C22—H22B	0.9700	O10—H10A	0.88 (4)
C23—C24	1.363 (3)	O10—H10B	0.85 (4)
			
C4—N1—C5	108.61 (19)	C21—C22—H22B	108.8
C4—N1—H1N	125.7	H22A—C22—H22B	107.7
C5—N1—H1N	125.7	C24—C23—C30	106.3 (2)
C1—N2—H2AN	109.5	C24—C23—C22	127.0 (2)
C1—N2—H2BN	109.5	C30—C23—C22	126.8 (2)
H2AN—N2—H2BN	109.5	C23—C24—N5	110.4 (2)
C1—N2—H2CN	109.5	C23—C24—H24	124.8
H2AN—N2—H2CN	109.5	N5—C24—H24	124.8
H2BN—N2—H2CN	109.5	N5—C25—C26	130.7 (2)
N2—C1—C2	111.8 (2)	N5—C25—C30	107.7 (2)
N2—C1—H1A	109.3	C26—C25—C30	121.6 (2)
C2—C1—H1A	109.3	C27—C26—C25	118.1 (3)
N2—C1—H1B	109.3	C27—C26—H26	121.0
C2—C1—H1B	109.3	C25—C26—H26	121.0
H1A—C1—H1B	107.9	C26—C27—C28	121.2 (3)
C3—C2—C1	114.13 (19)	C26—C27—H27	119.4
C3—C2—H2A	108.7	C28—C27—H27	119.4
C1—C2—H2A	108.7	C29—C28—C27	121.3 (3)
C3—C2—H2B	108.7	C29—C28—H28	119.4
C1—C2—H2B	108.7	C27—C28—H28	119.4
H2A—C2—H2B	107.6	C28—C29—C30	118.8 (2)
C4—C3—C10	106.3 (2)	C28—C29—H29	120.6
C4—C3—C2	126.1 (2)	C30—C29—H29	120.6
C10—C3—C2	127.6 (2)	C29—C30—C25	119.0 (2)
C3—C4—N1	110.9 (2)	C29—C30—C23	134.1 (2)
C3—C4—H4	124.6	C25—C30—C23	106.8 (2)
N1—C4—H4	124.6	C35—N7—C34	108.97 (19)
N1—C5—C6	130.8 (2)	C35—N7—H7N	125.5
N1—C5—C10	107.4 (2)	C34—N7—H7N	125.5
C6—C5—C10	121.8 (2)	C31—N8—H8AN	109.5
C7—C6—C5	117.9 (2)	C31—N8—H8BN	109.5
C7—C6—H6	121.1	H8AN—N8—H8BN	109.5
C5—C6—H6	121.1	C31—N8—H8CN	109.5
C6—C7—C8	121.2 (3)	H8AN—N8—H8CN	109.5
C6—C7—H7	119.4	H8BN—N8—H8CN	109.5
C8—C7—H7	119.4	N8—C31—C32	111.9 (2)
C9—C8—C7	121.2 (3)	N8—C31—H31A	109.2
C9—C8—H8	119.4	C32—C31—H31A	109.2
C7—C8—H8	119.4	N8—C31—H31B	109.2
C8—C9—C10	119.3 (2)	C32—C31—H31B	109.2
C8—C9—H9	120.4	H31A—C31—H31B	107.9
C10—C9—H9	120.4	C33—C32—C31	114.23 (19)
C9—C10—C5	118.6 (2)	C33—C32—H32A	108.7
C9—C10—C3	134.5 (2)	C31—C32—H32A	108.7
C5—C10—C3	106.8 (2)	C33—C32—H32B	108.7
C15—N3—C14	108.9 (2)	C31—C32—H32B	108.7
C15—N3—H3N	125.5	H32A—C32—H32B	107.6
C14—N3—H3N	125.5	C34—C33—C40	106.2 (2)
C11—N4—H4AN	109.5	C34—C33—C32	126.0 (2)
C11—N4—H4BN	109.5	C40—C33—C32	127.7 (2)
H4AN—N4—H4BN	109.5	C33—C34—N7	110.3 (2)
C11—N4—H4CN	109.5	C33—C34—H34	124.8
H4AN—N4—H4CN	109.5	N7—C34—H34	124.8
H4BN—N4—H4CN	109.5	N7—C35—C36	130.6 (2)
N4—C11—C12	111.9 (2)	N7—C35—C40	107.7 (2)
N4—C11—H11A	109.2	C36—C35—C40	121.7 (2)
C12—C11—H11A	109.2	C37—C36—C35	117.8 (2)
N4—C11—H11B	109.2	C37—C36—H36	121.1
C12—C11—H11B	109.2	C35—C36—H36	121.1
H11A—C11—H11B	107.9	C36—C37—C38	121.2 (3)
C13—C12—C11	113.95 (19)	C36—C37—H37	119.4
C13—C12—H12A	108.8	C38—C37—H37	119.4
C11—C12—H12A	108.8	C39—C38—C37	121.3 (3)
C13—C12—H12B	108.8	C39—C38—H38	119.3
C11—C12—H12B	108.8	C37—C38—H38	119.3
H12A—C12—H12B	107.7	C38—C39—C40	119.3 (2)
C14—C13—C20	106.3 (2)	C38—C39—H39	120.4
C14—C13—C12	126.7 (2)	C40—C39—H39	120.4
C20—C13—C12	127.0 (2)	C39—C40—C35	118.8 (2)
C13—C14—N3	110.3 (2)	C39—C40—C33	134.4 (2)
C13—C14—H14	124.8	C35—C40—C33	106.7 (2)
N3—C14—H14	124.8	O1—C41—O2	122.8 (2)
N3—C15—C16	130.4 (2)	O1—C41—C42	118.7 (2)
N3—C15—C20	107.6 (2)	O2—C41—C42	118.5 (2)
C16—C15—C20	122.0 (2)	C41—C42—H42A	109.5
C17—C16—C15	117.3 (3)	C41—C42—H42B	109.5
C17—C16—H16	121.3	H42A—C42—H42B	109.5
C15—C16—H16	121.3	C41—C42—H42C	109.5
C16—C17—C18	121.7 (3)	H42A—C42—H42C	109.5
C16—C17—H17	119.2	H42B—C42—H42C	109.5
C18—C17—H17	119.2	O3—C43—O4	124.2 (2)
C19—C18—C17	121.1 (3)	O3—C43—C44	117.6 (2)
C19—C18—H18	119.4	O4—C43—C44	118.1 (2)
C17—C18—H18	119.4	C43—C44—H44A	109.5
C18—C19—C20	119.5 (3)	C43—C44—H44B	109.5
C18—C19—H19	120.2	H44A—C44—H44B	109.5
C20—C19—H19	120.2	C43—C44—H44C	109.5
C19—C20—C15	118.4 (2)	H44A—C44—H44C	109.5
C19—C20—C13	134.8 (2)	H44B—C44—H44C	109.5
C15—C20—C13	106.8 (2)	O5—C45—O6	123.2 (2)
C25—N5—C24	108.81 (19)	O5—C45—C46	118.9 (2)
C25—N5—H5N	125.6	O6—C45—C46	117.9 (2)
C24—N5—H5N	125.6	C45—C46—H46A	109.5
C21—N6—H6AN	109.5	C45—C46—H46B	109.5
C21—N6—H6BN	109.5	H46A—C46—H46B	109.5
H6AN—N6—H6BN	109.5	C45—C46—H46C	109.5
C21—N6—H6CN	109.5	H46A—C46—H46C	109.5
H6AN—N6—H6CN	109.5	H46B—C46—H46C	109.5
H6BN—N6—H6CN	109.5	O8—C47—O7	123.9 (2)
N6—C21—C22	111.64 (19)	O8—C47—C48	118.5 (2)
N6—C21—H21A	109.3	O7—C47—C48	117.63 (19)
C22—C21—H21A	109.3	C47—C48—H48A	109.5
N6—C21—H21B	109.3	C47—C48—H48B	109.5
C22—C21—H21B	109.3	H48A—C48—H48B	109.5
H21A—C21—H21B	108.0	C47—C48—H48C	109.5
C23—C22—C21	113.92 (19)	H48A—C48—H48C	109.5
C23—C22—H22A	108.8	H48B—C48—H48C	109.5
C21—C22—H22A	108.8	H9A—O9—H9B	105 (4)
C23—C22—H22B	108.8	H10A—O10—H10B	102 (3)
			
N2—C1—C2—C3	−58.5 (3)	N6—C21—C22—C23	−64.6 (3)
C1—C2—C3—C4	103.1 (3)	C21—C22—C23—C24	100.7 (3)
C1—C2—C3—C10	−75.4 (3)	C21—C22—C23—C30	−78.6 (3)
C10—C3—C4—N1	−0.3 (3)	C30—C23—C24—N5	−0.5 (3)
C2—C3—C4—N1	−179.0 (2)	C22—C23—C24—N5	−179.8 (2)
C5—N1—C4—C3	0.0 (3)	C25—N5—C24—C23	−0.1 (3)
C4—N1—C5—C6	−179.4 (2)	C24—N5—C25—C26	−179.2 (3)
C4—N1—C5—C10	0.2 (3)	C24—N5—C25—C30	0.6 (3)
N1—C5—C6—C7	179.5 (2)	N5—C25—C26—C27	179.2 (3)
C10—C5—C6—C7	−0.1 (4)	C30—C25—C26—C27	−0.6 (4)
C5—C6—C7—C8	−0.9 (4)	C25—C26—C27—C28	−0.3 (4)
C6—C7—C8—C9	1.2 (4)	C26—C27—C28—C29	0.7 (5)
C7—C8—C9—C10	−0.4 (4)	C27—C28—C29—C30	−0.2 (4)
C8—C9—C10—C5	−0.7 (4)	C28—C29—C30—C25	−0.7 (4)
C8—C9—C10—C3	−178.5 (3)	C28—C29—C30—C23	−177.9 (3)
N1—C5—C10—C9	−178.8 (2)	N5—C25—C30—C29	−178.7 (2)
C6—C5—C10—C9	0.9 (3)	C26—C25—C30—C29	1.1 (4)
N1—C5—C10—C3	−0.4 (2)	N5—C25—C30—C23	−0.9 (3)
C6—C5—C10—C3	179.3 (2)	C26—C25—C30—C23	179.0 (2)
C4—C3—C10—C9	178.4 (3)	C24—C23—C30—C29	178.2 (3)
C2—C3—C10—C9	−2.9 (4)	C22—C23—C30—C29	−2.4 (4)
C4—C3—C10—C5	0.4 (2)	C24—C23—C30—C25	0.8 (3)
C2—C3—C10—C5	179.1 (2)	C22—C23—C30—C25	−179.8 (2)
N4—C11—C12—C13	59.5 (3)	N8—C31—C32—C33	−56.0 (3)
C11—C12—C13—C14	−102.8 (3)	C31—C32—C33—C34	103.2 (3)
C11—C12—C13—C20	78.1 (3)	C31—C32—C33—C40	−76.4 (3)
C20—C13—C14—N3	0.6 (3)	C40—C33—C34—N7	−0.8 (3)
C12—C13—C14—N3	−178.7 (2)	C32—C33—C34—N7	179.5 (2)
C15—N3—C14—C13	−0.8 (3)	C35—N7—C34—C33	0.9 (3)
C14—N3—C15—C16	179.9 (2)	C34—N7—C35—C36	179.4 (2)
C14—N3—C15—C20	0.6 (3)	C34—N7—C35—C40	−0.5 (3)
N3—C15—C16—C17	−178.2 (3)	N7—C35—C36—C37	178.8 (3)
C20—C15—C16—C17	1.0 (4)	C40—C35—C36—C37	−1.3 (4)
C15—C16—C17—C18	−0.3 (4)	C35—C36—C37—C38	0.3 (4)
C16—C17—C18—C19	−0.3 (5)	C36—C37—C38—C39	0.3 (5)
C17—C18—C19—C20	0.2 (4)	C37—C38—C39—C40	0.1 (4)
C18—C19—C20—C15	0.5 (4)	C38—C39—C40—C35	−1.1 (4)
C18—C19—C20—C13	178.5 (3)	C38—C39—C40—C33	−178.9 (3)
N3—C15—C20—C19	178.3 (2)	N7—C35—C40—C39	−178.4 (2)
C16—C15—C20—C19	−1.1 (3)	C36—C35—C40—C39	1.7 (3)
N3—C15—C20—C13	−0.3 (2)	N7—C35—C40—C33	0.0 (2)
C16—C15—C20—C13	−179.6 (2)	C36—C35—C40—C33	−179.9 (2)
C14—C13—C20—C19	−178.4 (3)	C34—C33—C40—C39	178.5 (3)
C12—C13—C20—C19	0.9 (4)	C32—C33—C40—C39	−1.8 (4)
C14—C13—C20—C15	−0.2 (2)	C34—C33—C40—C35	0.5 (2)
C12—C13—C20—C15	179.1 (2)	C32—C33—C40—C35	−179.8 (2)

### Hydrogen-bond geometry (Å, º)

Cg2, Cg5, Cg8 and Cg11 are the centroids of the benzene rings C5-C10, C15-C20, C25-C30 and C35-C40, respectively. Cg3, Cg6, Cg9 and Cg12 are the centroids of the indole ring systems N1/C3-C10, N3/C13-C20, N5/C23-C30 and N7/C33-C40, respectively.

**Table:**

*D*—H···*A*	*D*—H	H···*A*	*D*···*A*	*D*—H···*A*
C42—H42*B*···*Cg*2	0.96	2.87	3.621 (3)	135
C44—H44*B*···*Cg*5	0.96	2.74	3.550 (3)	143
C46—H46*B*···*Cg*8	0.96	2.80	3.533 (3)	134
C48—H48*B*···*Cg*11	0.96	2.78	3.629 (3)	147
N1—H1*N*···O4^i^	0.86	2.08	2.898 (2)	159
N2—H2*AN*···O6	0.89	2.02	2.861 (3)	156
N2—H2*BN*···O8	0.89	1.93	2.778 (2)	158
N2—H2*CN*···O1	0.89	1.92	2.803 (2)	169
N3—H3*N*···O2^ii^	0.86	2.04	2.864 (3)	161
N4—H4*AN*···O4	0.89	2.03	2.805 (3)	145
N4—H4*BN*···O5^i^	0.89	1.91	2.777 (2)	163
N4—H4*CN*···O7^ii^	0.89	2.45	3.186 (3)	140
N5—H5*N*···O7^iii^	0.86	2.01	2.839 (2)	162
N6—H6*AN*···O4^i^	0.89	2.57	3.122 (3)	121
N6—H6*BN*···O1	0.89	1.95	2.828 (2)	169
N6—H6*CN*···O5	0.89	2.07	2.936 (3)	165
N7—H7*N*···O6	0.86	2.04	2.867 (3)	161
N8—H8*AN*···O2	0.89	2.09	2.936 (3)	157
N8—H8*BN*···O3^ii^	0.89	1.85	2.734 (2)	172
N8—H8*CN*···O7	0.89	1.87	2.726 (2)	162
C4—H4···O5	0.93	2.40	3.248 (3)	151
C34—H34···O1	0.93	2.46	3.347 (3)	159
N4—H4*CN*···O9^iv^	0.89	2.46	3.003 (3)	120
O9—H9*A*···O8	0.88 (5)	1.97 (5)	2.840 (3)	169 (4)
O9—H9*B*···O6	0.86 (4)	2.02 (4)	2.872 (3)	168 (4)
N6—H6*AN*···O10	0.89	2.22	2.927 (3)	136
O10—H10*A*···O3	0.88 (4)	1.96 (4)	2.822 (3)	166 (3)
O10—H10*B*···O2^ii^	0.85 (4)	2.07 (4)	2.903 (3)	169 (3)
C9—H9···*Cg*12^v^	0.93	2.93	3.782 (3)	153
C19—H19···*Cg*9^vi^	0.93	2.81	3.641 (3)	149
C29—H29···*Cg*3^vii^	0.93	2.92	3.736 (3)	147
C37—H37···*Cg*6^viii^	0.93	2.83	3.692 (3)	155

Symmetry codes: (i) −*x*+1, −*y*, −*z*+1; (ii) −*x*+1, −*y*+1, −*z*+1; (iii) *x*, *y*−1, *z*; (iv) *x*, *y*, *z*+1; (v) *x*+1, *y*, *z*; (vi) −*x*, −*y*, −*z*+1; (vii) *x*−1, *y*, *z*; (viii) −*x*, −*y*+1, −*z*+1.
