# Crystal structure and Hirshfeld surface analysis of 2-(1*H*-indol-3-yl)ethanaminium acetate hemihydrate. Corrigendum

**DOI:** 10.1107/S2056989020012815

**Published:** 2020-09-30

**Authors:** Balakrishnan Rajeswari, Radhakrishnan Santhi, Palaniyappan Sivajeyanthi, Kasthuri Balasubramani

**Affiliations:** a PG and Research Department of Chemistry, Seethalakshmi Ramaswamy College (Affiliated to Bharathidasan University), Tiruchirappalli-2, Tamil Nadu, India; b Department of Chemistry, Government Arts College (Affiliated to Bharathidasan University), Thanthonimalai, Karur 639 005, Tamil Nadu, India

## Abstract

In the paper by Rajeswari *et al.* [*Acta Cryst.* (2019), E**75**, 451–455], the affiliations were not given correctly.

## Abstract 2

Corrigendum to *Acta Cryst.* (2019), E**75**, 451–455.

The affiliations in the paper by Rajeswari *et al.* (2019 ▸) are incorrect and should be ‘^a^PG and Research Department of Chemistry, Seethalakshmi Ramaswamy College (Affiliated to Bharathidasan University), Tiruchirappalli-2, Tamil Nadu, India and ^b^Department of Chemistry, Government Arts College (Affiliated to Bharathidasan University), Thanthonimalai, Karur 639 005, Tamil Nadu, India’, as given above.
